# Efficient and Interpretable Prediction of Protein Functional Classes by Correspondence Analysis and Compact Set Relations

**DOI:** 10.1371/journal.pone.0075542

**Published:** 2013-10-11

**Authors:** Jia-Ming Chang, Jean-Francois Taly, Ionas Erb, Ting-Yi Sung, Wen-Lian Hsu, Chuan Yi Tang, Cedric Notredame, Emily Chia-Yu Su

**Affiliations:** 1 Comparative Bioinformatics, Bioinformatics and Genomics, Centre for Genomic Regulation (CRG), Barcelona, Spain; 2 Universitat Pompeu Fabra (UPF), Barcelona, Spain; 3 Bioinformatics Core Facility, Centre for Genomic Regulation (CRG), Barcelona, Spain; 4 Bioinformatics Lab., Institute of Information Science, Academia Sinica, Taipei, Taiwan; 5 Department of Computer Science, National Tsing Hua University, Hsinchu, Taiwan; 6 Department of Computer Science and Information Engineering, Providence University, Taichung, Taiwan; 7 Graduate Institute of Biomedical Informatics, College of Medical Science and Technology, Taipei Medical University, Taipei, Taiwan; University of Bonn, Bonn-Aachen International Center for IT, Germany

## Abstract

Predicting protein functional classes such as localization sites and modifications plays a crucial role in function annotation. Given a tremendous amount of sequence data yielded from high-throughput sequencing experiments, the need of efficient and interpretable prediction strategies has been rapidly amplified. Our previous approach for subcellular localization prediction, PSLDoc, archives high overall accuracy for Gram-negative bacteria. However, PSLDoc is computational intensive due to incorporation of homology extension in feature extraction and probabilistic latent semantic analysis in feature reduction. Besides, prediction results generated by support vector machines are accurate but generally difficult to interpret.

In this work, we incorporate three new techniques to improve efficiency and interpretability. First, homology extension is performed against a compact non-redundant database using a fast search model to reduce running time. Second, correspondence analysis (CA) is incorporated as an efficient feature reduction to generate a clear visual separation of different protein classes. Finally, functional classes are predicted by a combination of accurate compact set (CS) relation and interpretable one-nearest neighbor (*1*-NN) algorithm. Besides localization data sets, we also apply a human protein kinase set to validate generality of our proposed method.

Experiment results demonstrate that our method make accurate prediction in a more efficient and interpretable manner. First, homology extension using a fast search on a compact database can greatly accelerate traditional running time up to twenty-five times faster without sacrificing prediction performance. This suggests that computational costs of many other predictors that also incorporate homology information can be largely reduced. In addition, CA can not only efficiently identify discriminative features but also provide a clear visualization of different functional classes. Moreover, predictions based on CS achieve 100% precision. When combined with *1*-NN on unpredicted targets by CS, our method attains slightly better or comparable performance compared with the state-of-the-art systems.

## Introduction

### 1. Background

In the post-genomic era, tremendous amounts of sequence data are generated from biological experiments. The increase in the number of putative protein sequences greatly exceeds that of known functions of proteins. Despite recent technical advances, experimental determination of protein function remains time-consuming and labor-intensive. Therefore, using computational approaches to extract functional information from sequences becomes an important issue for global analysis of biological systems.

Identification of protein functional classes plays a critical role in function annotation. For example, prediction of localization sites and modifications in a protein can help understand its biological functions and cellular mechanisms. Protein subcellular localization (PSL) prediction focuses on determining localization sites of unknown proteins in a cell. It provides an efficient way to depict protein functions and annotate newly sequenced genomes. Identification of PSL is also crucial to detect cell surface or secreted drug targets and biomarkers. In addition, protein kinases (PKs) consist of a key class of enzymes responsible of signal transduction in regulation pathways modifying the cell cycle in function of extracellular signals through reversible phosphorylation. The signal is sensed in the membrane and then transferred into the nucleus by an enzymatic activation cascade of the PKs, which are classified into subfamilies according to the amino acid sequences of their catalytic domains. Therefore, the studies of subcellular localization and kinase classification are important for elucidating protein functions involved in various cellular processes and signal transduction pathways. Moreover, they are crucial for the identification of drug targets and may serve as an important indicator of several diseases, like cancer and Alzheimer's disease.

### 2. Previous work

Many systems have been developed to analyze protein functional classes. For PSL prediction, the prediction systems can be generally classified into amino acid composition-based methods, homology-based methods, and methods that integrate various protein characteristics. The amino acid composition-based methods, such as CELLO [1], P-CLASSIFIER [2], WoLF Psort [3], TargetP [4], [5], PSLDoc [6], and PSLNuc [7], utilize different encoding schemes of protein sequences. Incorporation of amino acid templates has also been shown useful for PSL prediction [8]. As illustrated in several methods, including PSLpred [9], PSORTb [10], [11], and PSLDoc, evolutionary information extracted from homologous proteins can enhance prediction performance. Finally, previous studies including PSORTb and PSL101 [12] demonstrate that combination of various protein physicochemical properties can achieve better performance than each individual module.

PKs classification in superior eukaryotes is a difficult task as the genes encoding those proteins diverged from their common ancestor a very long time ago. However, the crucial function of this protein family forced all sequences to maintain common features insuring the stability of an active tertiary structure of the enzyme. PKs are thus a mixture of very conserved and divergent sequence portions. A good classifier would be able to distinguish informative signals from too conserved portions and background noise. This difficult task is often performed thanks to the use of hidden Markov models (HMM) based on accurate multiple sequence alignments (MSAs) of well characterized protein kinase families. Kinomer [13] is one the most successful PK classifier using this methodology. Based on the known classification of several PKs in model organisms, Kinomer sub-divided the groups into smaller families from which they derived accurate HMM.

### 3. Challenges

Although our previous prediction method, PSLDoc, attains high accuracy in PSL prediction, three disadvantages can be further improved. First, for feature extraction and representation, PSLDoc requires a huge amount of running time to generate position-specific scoring matrix (PSSM) in homology extension. The homology extension procedure has been intensively used in many bioinformatics software, i.e., PSIPRED [14], MEMSAT3 [15], RNAProB [16], PSI-Coffee [17], and etc. However, the effects of database sizes and sequence identities on prediction accuracy have not been fully investigated. The speed of a prediction procedure can be greatly increased if the running time of homology extension is reduced by searching against a smaller database without sacrificing prediction accuracy. Second, feature reduction in PSLDoc performed by probabilistic latent semantic analysis (PLSA) [18] is computationally intensive and inefficient. PSLDoc uses PLSA to reduce features based on mappings created by latent semantic topics for each protein localization class. However, PLSA requires a huge computational cost and long running time for model-fitting by expectation-maximization (EM) algorithm. Even with a trained model, we still need to combine different information before identifying the final signature relation for testing (Figure 8, 9, and 10 in [6]). Finally, prediction results generated by support vector machines (SVM) in PSLDoc could not be easily interpreted. Like many other prediction systems, PSLDoc utilizes SVM to learn a model and perform accurate prediction, but it is relatively difficult to interpret prediction results from SVM. When compared with simple *k*-nearest neighbor (*k*-NN) or decision tree algorithms, SVM suffers from the problem of low data interpretability due to the lack of interpretable closest training examples or decision rules.

### 4. Our contributions

To overcome the above shortcomings, we proposed a new prediction strategy in which incorporates a faster homology extension procedure as feature extraction, correspondence analysis (CA) as an efficient feature reduction approach, and compact set relations with nearest neighbor algorithm as an interpretable prediction method. First, the running time and computational cost of homology extension can be greatly reduced using a fast search on a smaller compact non-redundant database. We investigate the running time and prediction accuracy for homology extension procedures using different settings and databases filtered at various sequence identities. Experiment results demonstrate that our method is twenty-five times faster than the traditional default setting by searching against a smaller compact database. Second, incorporation of CA for feature reduction is less computationally intensive compared to PLSA. Our analysis demonstrates that CA can identify discriminative features from protein sequences in a more efficient manner. Moreover, after performing CA, it is also intuitive to observe that protein sequences in the same functional classes tend to cluster closer than those in different classes. Finally, combination of compact sets with *k*-NN for prediction can provide a more interpretable visualization of protein sequences and their corresponding functional classes. Applying compact set relations to predict functional classes can generate highly reliable prediction results. In addition, *1*-NN can be considered as the simplest classification approach in *k*-NN algorithms without tuning the parameter *k*. Based on the above observations, we combine compact set relations with *1*-NN algorithm to achieve high prediction accuracy without sacrificing data interpretability.

## Materials and Methods

In this study, we propose a method to predict protein functional classes based on CA and compact sets. First, gapped-dipeptides are extracted from a protein sequence and weighted by evolutionary information from homology extension. Then, CA is conducted to reduce the feature dimension of gapped-dipeptides. After that, compact set relations are identified. Finally, the prediction is made by compact sets if the target protein belongs to part of the sets; otherwise, a one-nearest neighbor is incorporated for the classification. The details of framework of PSLDoc system, feature representation, feature reduction, functional class prediction, system architecture, data sets, and evaluation measures are described in the following sections.

### 1. Framework of PSLDoc

We proposed PSLDoc for protein localization prediction based on document classification techniques. First, sequence features of a protein sequence are extracted from gapped-dipeptides, which are composed of pairs of amino acids separated by various gap distances. If a protein sequence is regarded as a document, the gapped-dipeptides within the sequence can be treated as the terms of the document. Then, evolutionary information from PSSM is incorporated to determine the weight of each gapped-dipeptide, which is referred to as TFPSSM scoring scheme and calculated from the term frequency of a document in the field of text mining [6]. After that, PLSA is incorporated for feature reduction and finally SVM is applied to predict a localization class based on the reduced features. Although PSLDoc can attain high prediction accuracy, it still suffers from the problems of low data interpretability and high computational costs.

### 2. Feature representation by gapped-dipeptides weighted by a new efficient homology extension approach

We apply the same feature representation from PSLDoc and investigate the effect of different database sizes and search models in homology extension. Each protein is first represented by a feature vector according to gapped-dipeptides and TFPSSM weighting scheme. Then gapped-dipeptides are extracted as features of a protein and their weights are determined according to evolutionary information from PSSM generated by PSI-BLAST [19]. However, the time needed to obtain evolutionary information by a homology extension procedure is positively correlated with the size of the searched database and could be tremendous. It has also been shown that highly non-redundant databases can be used to obtain similar sequence alignment accuracy with a significantly reduced computational cost [20]. Here we compare the influence of a database on protein classification accuracy by searching against different databases, including NCBI (National Center for Biotechnology Information) nr, UniProt, and several UniRef non-redundant databases (i.e., UniRef100, UniRef90, and UniRef50) [21]. For the UniRef databases, a database is filtered so as to make certain that no pair of sequences exists with an identity higher than a specific level. For example, UniRef50 database is trimmed from the UniProt database to ensure no pair of sequences shares higher than 50% sequence identity. Besides the size of a database, the parameter settings of PSI-BLAST are also analyzed. Two parameter settings are benchmarked. One is regarded as a *normal* model, with iteration number as two and e-value threshold at 0.001, and the other is considered *fast* (insensitive) search suggested by BLAST [22] with soft masking, using BLOSUM80 matrix, neighborhood word threshold score as 999, gap open penalty as 9, gap extend as 2, and e-value threshold at 1e-5 (The corresponding parameter of NCBI+ is “–matrix BLOSUM80 –evalue 1e-5 –gapopen 9 –gapextend 2 –threshold 999 – seq yes –soft_masking true –numter_iteration 2”) [22].

### 3. Feature reduction by a more efficient and intuitive correspondence analysis

PSLDoc introduced PLSA to detect the preference relation between gapped-dipeptides and protein localization classes, that is, certain gapped-dipeptides highly frequently exist in a protein localization class (Table 7 in [6]). PLSA uses a latent variable model for co-occurrence data (i.e., documents and terms) in which each observation is associated with an unobserved class variable. The parameters of the PLSA model are then estimated by an iterative EM learning process, which is extremely computational intense. Furthermore, even with a trained model, the gapped-dipeptide signatures frequently observed in each protein class can be identified through combining both *P*(*w*|*z*), the topic-conditional probability of a term condition on the unobserved topic, and *P*(*z*|*d*), a document-specific probability distribution over the latent variable space (Figure 8, 9, and 10 in [6]). To improve the efficiency in feature reduction, we propose an efficient CA, which can effectively project gapped-dipeptides and proteins into the same reduced dimensional space such that we can identify the preference between gapped-dipeptides and protein sequences in a more intuitive and visible way.

CA is generally used to explore the dependencies that exist between a number of observations and a set of categories that define them. It is an exploratory multivariate technique that converts frequency table data into graphical displays in which rows and columns are depicted as points. In our case, a protein is characterized by several gapped-dipeptides of a certain kind, and CA can reveal the correspondences between the gapped-dipeptide variables and the protein classes, which our observations fall in. A thorough account of the technique is given in the book by Greenacre [23]. The row profiles are the frequencies in the rows divided by their row sums. CA is a generalized principal coordinates analysis (PCA) of the row profiles and a generalized PCA of the columns profiles, and the treatment of the rows and columns is the same. Both the row and column profiles rely on the same matrix decomposition as follows (adopted from [24]):

1. Divide the original data table *N* by its grand total *n*:

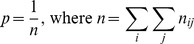

1. Denote by *r* and *c* the marginal sums of *P*: *r* = *P*1, *c* = *P* ^T^1
2. Calculate the matrix of standardized residuals and its SVD:

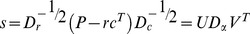

1. Calculate the coordinates:

Principal coordinates of 
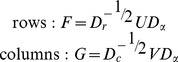


Roughly, an observation is described as a vector over its variables, and a variable is represented by a vector over its observations. Centering and normalizing of the data matrix has the effect that these vectors can be seen as discrete probability distributions, and distances within each class of vectors are interpreted as the well-known Chi-squared distance. In fact, CA decomposes the Chi-squared statistic of the joint distribution of variables and observations from its independent model of uncorrelated variables and observations. In a typical CA plot, each axis (i.e., as in PCA) reflects a certain amount of the total variance in the data. Interestingly, both variables and observations can be plotted in this coordinate system, and the variables and observations contributing most to the total variance will typically fall far away from the origin of the plot (which represents the independent model). This way, correspondences and their main drivers can be studied in an intuitive way. CA has been shown useful in predicting functional residues in proteins [25], [26]. In this work, CA is conducted using the R-package, FactoMineR [27]. The sizes of reduced dimensions are chosen such that these factors explain more the 95% variance of the data together (detailed later in Table 1 of the Results section).

**Table 1:** Performance comparison of fast search and normal search on databases of different sizes for the GramNeg_1444 data set.

			Fast search (Normal search*)		
Database	# of seq.	Time (sec.)	Accuracy (%)	# of CA dim.	Ave. IPP
UniRef50	3,077,464	21,219 (139,667)	90.10 (90.34)	29 (21)	0.742 (0.607)
UniRef90	6,544,144	44,999 (315,518)	89.89 (90.03)	37 (29)	0.817 (0.699)
UniRef100	9,865,668	67,797 (496,425)	90.10 (90.10)	44 (36)	0.857 (0.749)
UniProt	11,009,767	73,623 (543,240)	90.58 (89.96)	52 (41)	0.888 (0.777)
NCBI nr	10,565,004	73,811 (536,104)	90.17 (90.44)	44 (36)	0.856 (0.749)

* Experiment results based on normal search are shown in parentheses.

### 4. Functional class prediction by combining compact sets and nearest neighbor algorithm

To predict functional classes of a protein, we first incorporate compact sets to analyze relations between functional classes and gapped-dipeptides. If a target sequence belongs to compact sets, we can make a confident prediction through the unambiguous majority voting of the other proteins in the largest compact set. However, not all of the protein sequences will be part of a compact set, and the sequences that do not belong to any compact set will be predicted by one-nearest neighbor (*1*-NN, i.e., *k* = 1 for *k*-NN) algorithm. In our study, the predictions made only by compact sets are denoted as CS, and the others determined by both compact sets and *1*-NN are represented by CS+*1*−NN. The details of compact sets and *k*-nearest neighbor algorithm are explained as follows.

#### 4.1 Compact sets

Given a fully connected undirected graph *G* = (*V*, *E*) in which *v_i_* 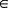 *V* represents a protein and the edge *E*(*v_i_*, *v_j_*) denotes the distance between two proteins *v_i_* and *v_j_* measured as the Euclidean distance of their corresponding vectors in CA reduced space. Therefore, the distance between any pair of proteins is known. For any subset *C* of *V*, *C* is called a *compact set* if the smallest external distance of *C* is still larger than the longest internal distance in *C*:

*C* is a compact set if *min*{*E*(*v_i_*,*v_k_*)|*v_i_* 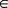 *C*,*v_k_* 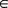 *V* \ *C*}> *max*{*D*(*v_i_*,*v_j_*)|*v_i_*,*v_j_* 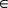 *C*}.

**Figure 1:**
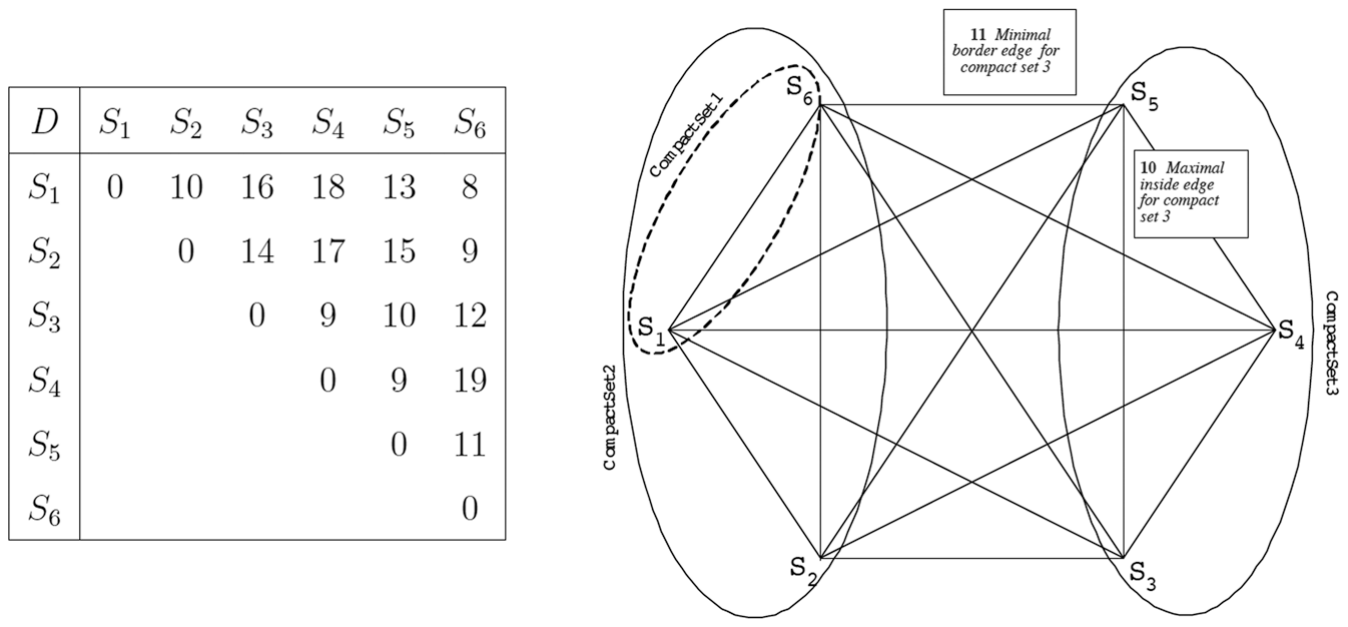
A compact set example. A distance matrix *D* for five sequences and its three nontrivial compact sets {*S*_1_, *S*_6_}, {*S*_1_, *S*_2_, *S*_6_}, and {*S*_3_, *S*_4_, *S*_5_}.

By definition, *V* is a compact set and each set consisting of single sequence is also a compact set (i.e., these compact sets are trivial). Considering the distance matrix of Figure 1 as an example, there are three nontrivial compact sets {*S*_1_, *S*_6_}, {*S*_1_, *S*_2_, *S*_6_}, and {*S*_3_, *S*_4_, *S*_5_} among the sequences. Nontrivial compact set can be considered as the strict neighboring relationship because sequences inside a compact set are closer to any one of them with a sequence from the outside of the set.

The first COMPACT_SET algorithm for identifying all compact sets in the graph *G* is provided by Zivkovic [28], which is similar to the Kruskal algorithm for finding a Minimum Spanning Tree (MST) (Section 23, [29]), merging edges one by one until all vertices are in one union set (|*E*| repeat-loop iterations). Then, Dekel, Hu, and Ouyang brought up a candidate tree *T_can_* of *G* which has a good property that each compact set of *G* is represented by a vertex in *T_can_* but not all vertices in *T_can_* are compact sets (Lemma 3.1, [30]). Kim presented an improved implementation of the above algorithm, assuming *G* is given as an adjacency list [31].

**Figure 2:**
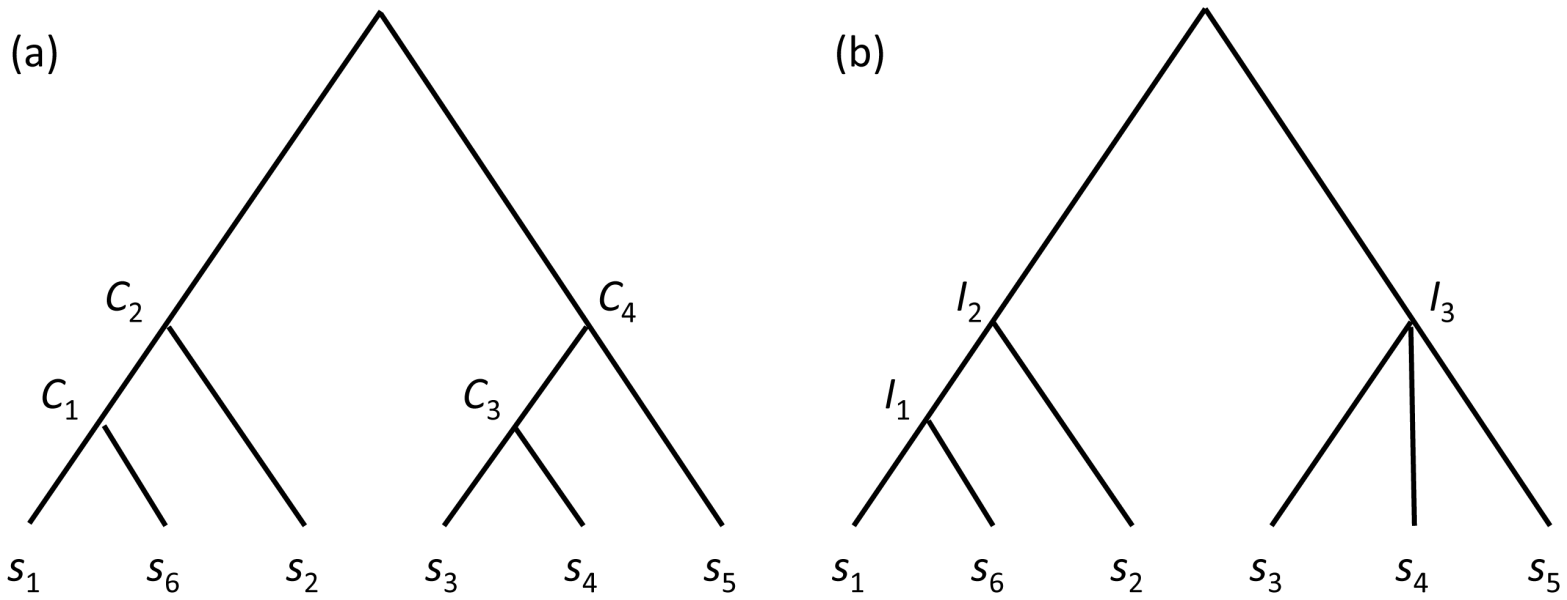
Kruskal Merging Ordering Tree *T_Kru_* and its compact set tree *T_c_*. (a) A Kruskal Merging Ordering Tree *T_Kru_* of the distance matrix *D* in Figure 1 with four internal nodes, *C_1_*, *C_2_*, *C_3_*, and *C_4_*, representing four candidate compact sets {*S*_1_, *S*_6_}, {*S*_1_, *S*_2_, *S*_6_}, {*S*_3_, *S*_4_} and {*S*_3_, *S*_4_, *S*_5_}, respectively. (b) Its compact set tree *T_c_* with three internal nodes, *I_1_*, *I_2_*, and *I_3_*, representing nontrivial compact sets {*S*_1_, *S*_6_}, {*S*_1_, *S*_2_, *S*_6_}, and {*S*_3_, *S*_4_, *S*_5_}, respectively.

Combining the Kruskal-like method in [28] with the properties of the *T_can_* tree in previous studies [30], [31], we proposed a Kruskal Merging Ordering Tree, *T_Kru_* (a new method to construct *T_can_*) such that identifying compact sets can be done in two steps shown as below [32], [33]. An example of *T_Kru_* ( = *T_can_*) and its corresponding compact tree, *T_c_*, are illustrated in Figure 2.

The compact set tree is a hierarchical clustering approach. Its backbone topology is *T_Kru_*, which is identical to a hierarchical tree created by Single-Linkage Clustering (SLC). However, a cluster defined by the internal node of a SLC hierarchical tree might not satisfy the compact set property. In the compact set method, this internal node will be merged within its parent in *T_c_* (i.e., Step 2 in the COMPACT_SET algorithm). Therefore, *T_c_* is not necessarily binary, but a SLC hierarchical tree is. A cluster created by the internal node of a SLC hierarchical tree possibly does not belong to clusters defined by *T_c_*. Conversely, clusters generated by *T_c_* are subsets of clusters built by SLC.

- **Algorithm**: COMPACT_SET
- **Input**: A weighted undirected fully connected graph *G*
- **Output**: Find all the compact sets in *G*
  -   Step 1. Construct a Kruskal Merging Ordering Tree   *T_Kru_* of *G*. (CONSTRUCT_*T_Kru_*)
  -   Step 2. Verify all candidate sets.
- **Algorithm**: CONSTRUCT_*T_Kru_*
- **Input**: *G*  =  (*V*, *E*), where |*V*|  =  *N* and |*E*|  =  *M*
- **Output**: Kruskal Merging Ordering Tree *T_Kru_*
- **begin**
- Step *a* **for** each vertex *v* 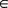 *V* **do**
  -        Make-Set (*v*);
  -    Sort the edges of *E* in a non-decreasing order;
  - **   for** *k* = 1 **to** *M*, *e_k_* = (*u*,*v*) 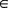 *E_s_* **do**
  - **      if** Find-Set (*u*) ! =  Find-Set (*v*) **then**
  -       Union (*u*, *v*);
- Step *b*     Merge the trees *T_u_* and *T_v_* into the new        tree *T_k_*;
  - **return** *T_k_*;
- **end**

We proceed with the detailed description of each step. The CONSTRUCT_*T_Kru_* algorithm in step 1 is merging edges one by one in the process of the Kruskal MST algorithm (Section 23, [29]). Initially, we create a rooted tree *T_i_* which contains only a node *v_i_* as its root for each vertex *v_i_* 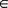 *V*. We sort the edges of *E* in a non-decreasing order such that we get the result *E* = {*e_1_*, *e_2_*,., *e_M_*} with *w*(*e_1_*) ≤ *w*(*e_2_*) ≤.≤ *w*(*e_M_*). For each edge *e_k_* = (*u*,*v*) 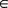 *E*,1≤ *k* ≤M, we find the trees *T_u_* and *T_v_* containing *u* and *v*, respectively, and then merge them into a new tree *T_k_* rooted at a new node *k* such that *u* and *v* become the children of *k* (step *b*). One nice property is that *w*(*e_k_*) is the minimum external distance for both groups *u* and *v* [30], [31]. The merging step is continued until only one tree remains, and it is *T_Kru_* which is identical to *T_can_* (Proof in Theorem 1, see Text S1 in Supporting Information). The original Kruskal MST algorithm does not have steps *a* (i.e., making sets for each vertex) and *b* (i.e., merging trees into a new tree). Step *a* can be done in *O*(*N*) and step *b* can be performed in *O*(1). Therefore, the time complexity of CONSTRUCT_*T_Kru_* is the same with that of Kruskal MST, *O*(*M* log *N*) (Section 23, [29]), where *M* and *N* are the numbers of edges and vertices in an edge-weighted graph.

For step 2 of the COMPACT_SET algorithm, we use the least common ancestor algorithm [31] to verify all candidate sets in *O*(*M* + *N*) time [34]. In conclusion, compact sets can be found in *O*(*L* + *M* + *M*log*N*) time, where *L* is the sum of the sizes of all compact sets.

#### 4.2 k-nearest neighbor algorithm

An unknown target is projected through CA as a point in the same dimension space with the training data set. Then, we calculate its Euclidean distance with respect to all training data, and the predicted class will be assigned with the same label as the nearest neighbor sequence in *1*-NN algorithm. Although simple *1*-NN usually does not predict as accurately as SVM, it not only performs efficiently but also generates more interpretive prediction results from the nearest neighbor relation compared with SVM. However, the lack of prediction confidence estimation in *k*-NN makes it difficult for the users to judge whether a prediction result is reliable or not. In addition, the *k*-NN algorithm is sensitive to the local structure of the data, that it, some cases can only be correctly predicted by *1*-NN, some only by *3*-NN, and some only by *5*-NN. Therefore, one of the challenges in using *k*-NN algorithm is to determine how many nearest neighbors are enough for each individual case.

### 5. System architecture

**Figure 3:**
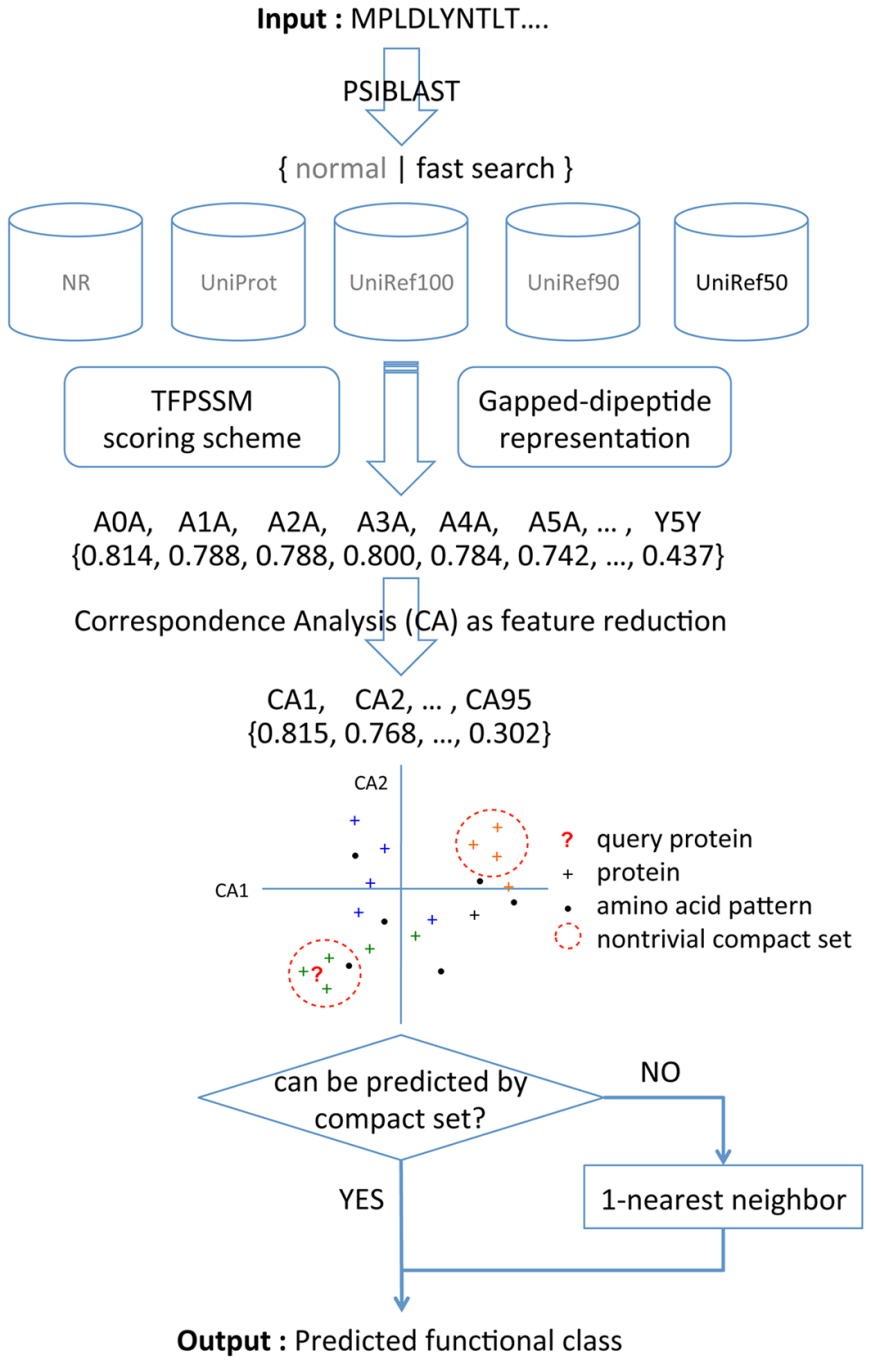
System architecture of the proposed method for prediction of protein functional classes. Using “fast search” on “UniRef50” database is the default setting of our method.

In this study, we present a protein functional class prediction method based on CA and homology extension. Our method incorporates gapped-dipeptides extracted from a protein sequence and weighted by homology extension information from PSSM. Next, CA is utilized as feature reduction to reduce the feature dimension of gapped-dipeptides. Then, a compact set relation analysis is performed. Finally, the protein is classified by CS or CS+*1*-NN. The system architecture of our method is shown in Figure 3. Given a protein sequence, our method performs the following steps:

1. Use PSI-BLAST to search against a non-redundant sequence database and generate homology extension information in PSSM
2. Extract gapped-dipeptides to represent the protein and assign weights according to TFPSSM scoring scheme
3. Apply CA for feature reduction
4. Generate protein gapped-dipeptide compact set relation based on reduced gapped-dipeptides
5. Incorporate CS+*1*−NN method to predict the protein functional class: if the target protein belongs to compact sets, make the confident prediction by the largest CS; otherwise, predict by *1*-NN.

### 6. Data sets

To demonstrate the generality of our method, both localization prediction and kinase classification are incorp*o*rated to evaluate prediction performance. For performance comparison with other approaches, we utilize protein localization and kinase classification data sets that have been used in previous studies.

#### 6.1 Protein subcellular localization data sets

For protein localization prediction, we utilize benchmark data sets curated in PSORTb 2.0 and PSORTb 3.0. First, to compare the performance of our new strategy with the original PSLDoc, we incorporate the Gram-negative bacteria benchmark data set (abbreviated as the GramNeg_1444 data set) organized in PSORTb 2.0, which consists of 1,444 experimentally determined proteins in five localization sites: cytoplasmic (CP), inner membrane (IM), periplasmic (PP), outer membrane (OM), and extracellular space (EC). The numbers of proteins distributed in each localization sites in the GramNeg_1444 data set are shown in Table 2. For comparison with the state-of-the-art system PSORTb 3.0, we also incorporate new benchmark data sets (abbreviated as the GramNeg_8230, GramPos_2652, and Archaeal_810 data sets in Table 3) built in PSORTb 3.0, which contain 8,230 Gram-negative, 2,652 Gram-positive, and 810 Archaeal proteins.

**Table 2:** Prediction performance of protein subcellular localization by five-fold cross-validation for the GramNeg_1444 data set.

		CS		CS+*1*−NN		PSLDoc	
Loc. Site	# of seq.	Precision (%)	Recall (%)	Precision (%)	Recall (%)	Precision (%)	Recall (%)
CP	278	**100.00**	46.40	92.34	88.85	91.03	**94.96**
IM	309	**100.00**	65.26	85.07	91.05	96.97	**93.20**
PP	76	**100.00**	55.02	96.22	**90.94**	89.13	89.13
OM	391	**100.00**	68.03	94.80	**97.95**	96.89	95.65
EC	190	**100.00**	65.58	88.15	88.41	87.69	**90.00**
Overall	1,444	**100.00**	60.25	91.97	91.97	93.01	**93.01**

The best performance of individual localization sites and overall data set is shown in bold face.

**Table 3:** Prediction performance of protein subcellular localization by five-fold cross-validation for the GramNeg_8230, GramPos_2652, and Archaeal_810 data sets.

Data set	Method	Precision (%)	Recall (%)	F-measure
GramNeg_8230	CS	**100.00**	51.50	0.6799
	*1*−NN	95.17	95.12	0.9514
	CS+*1*−NN	96.08	**96.04**	**0.9606**
	PSORTb 3.0*	97.30	94.10	0.9567
GramPos_2652	CS	**100.00**	59.20	0.7437
	*1*−NN	93.73	93.48	0.9361
	CS+*1*−NN	95.06	**94.84**	0.9495
	PSORTb 3.0*	98.20	93.10	**0.9558**
Archaeal_810	CS	**100.00**	69.74	0.8218
	*1*−NN	96.76	96.40	0.9658
	CS+*1*−NN	96.89	**96.89**	**0.9689**
	PSORTb 3.0*	97.20	93.40	0.9526

* Prediction performance of PSORTb 3.0 by five-fold cross-validation is obtained from Table 2 and Table 3 in [11].

The best performance of individual data sets is shown in bold face.

#### 6.2 Protein kinase classification data set

Although the original PSLDoc framework is designed to predict PSL, it can also be extended to other protein classification problems. To validate the generality of our method, we also analyze its prediction power on the annotation of human PKs. Since the first classification realized by Hanks and Hunter in 1995 [35], and one of its major updates in 2002 [36], the structures of the kinome classification have not been changed. The 516 known human kinases are distributed in eight families of eukaryotic protein kinases (ePKs) (i.e., AGC, CAMK, CK1, CMGC, RGC, STE, TK, and TKL) and different numbers of atypical protein kinases (aPKs) depending on the study. In this work, we focus our interest only on the ePKs as aPKs are often related to different functions and can be considered as an out-group. Our kinase data set, called hereafter the HumanKinase_409 set, is downloaded from KinBase database (http://kinase.com/kinbase/FastaFiles/Human_kinase_domain.fasta) and it contains 409 protein sequences classified into the eight families detailed in Table 4. To the best of our knowledge, we compare our results to the most recent classification method, Kinomer [13].

**Table 4:** Prediction performance of human kinase classification by five-fold cross-validation for the HumanKinase_409 data set.

		CS		CS+*1*−NN		Kinomer*
Family	# of seq.	Precision (%)	Recall (%)	Precision (%)	Recall (%)	Recall (%)
AGC	69	**100.00**	72.46	**100.00**	95.65	98.41
CAMK	76	**100.00**	73.68	**100.00**	**100.00**	**100.00**
CK1	12	**100.00**	83.33	92.31	**100.00**	**100.00**
CMGC	63	**100.00**	74.60	98.44	**100.00**	**100.00**
RGC	05	**100.00**	40.00	**100.00**	80.00	**100.00**
STE	47	**100.00**	89.36	**100.00**	**100.00**	**100.00**
TK	94	**100.00**	62.04	98.56	**100.00**	**100.00**
TKL	43	**100.00**	58.14	95.56	**100.00**	**100.00**
Overall	409	**100.00**	71.39	99.02	99.02	**99.77**

* Prediction performance of Kinomer, which was trained and tested on the same data set, is obtained from Table III in [13].

The best performance of individual kinase families and overall data set is shown in bold face.

### 7. Evaluation measures

To compare with other approaches, we follow the same evaluation measures used in previous studies [1], [6], [11], [13]. In our experiment, we use five-fold cross-validation to evaluate our prediction performance. First, a data set is randomly split into five disjoint parts of equal size. One of the five parts is used as a testing set, and the remaining parts are joined as a training set. A training set is used to get the coordinate system for CA projection and a testing set is projected to the same space as obtained from the training set according to the trained CA. A testing set is predicted based on CS or CS+*1*−NN. The procedure is iterated five times and each time a different part is chosen as a testing set. To assess the performance of each protein class, recall and precision are calculated by Equations (1) and (2), respectively. We also use accuracy defined in Equation (3) to assess the overall prediction performance and apply F-measure defined in Equation (4) to evaluate the harmonic mean of recall and precision. TP, FP, FN, and N denote the numbers of true positives, false positives, false negatives, and total number of proteins, respectively.
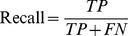
(1)

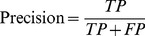
(2)

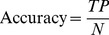
(3)

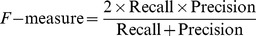
(4)


## Results

### 1. Compact non-redundant database achieves high prediction performance with much less running time

When we calculate the TFPSSM weighting scheme of gapped-dipeptides, evolutionary information of PSSM is generated by BLAST search for each protein against a database. This procedure defines the database as a key ingredient of homology extension. Therefore, it is an interesting question to ask how this parameter may affect the overall accuracy of the procedure. To investigate the effect of database sizes and search models, we incorporate *1*−NN on the GramNeg_1444 data set for comparison in this section. The information per position (IPP) provides a quantitative measure of sequence conservation among the homologous sequences used to construct the PSSM for each sequence position [37]. This value, which is stored in the second last column in PSI-BLAST profiles, is provided directly by PSI-BLAST. Table 1 demonstrates the prediction performance using fast search and normal search (shown in parentheses) on UniRef50, UniRef90, UniRef100, UniProt, and NCBI nr databases. We compare the effect of different database sizes and search models as following.

First, we compare prediction performance and running time among databases of different sizes. Experiment results show that the differences in accuracy are very small among those databases for both normal search and fast search. For example, the 90.10% accuracy of fast search against UniRef50 database is almost as good as 90.58% and 90.17% by UniProt and NCBI nr, respectively. Moreover, the numbers of CA dimension and the values of average IPP in our method also decrease when more redundant sequences are filtered in the searched databases. This implies that the incorporation of a more compact non-redundant database can achieve equal prediction performance with less running time compared to large databases. In fact, the UniRef50 database filters protein sequences at 50% redundancy and is approximately 3.5 times smaller than the traditionally searched UniProt and NCBI nr databases. It suggests that the central processing unit (CPU) requirements and computational time in homology extension could be greatly reduced by one-fourth faster (139,667 sec. vs. 543,240 sec.), without sacrificing prediction power based on a more compact non-redundant database.

Second, we compare prediction performance and running time of fast search and normal search. Interestingly, using fast insensitive search on UniRef50 database can further reduce 85% running time (21,219 sec. vs. 139,667 sec.) with almost identical accuracy (90.10% vs. 90.34%) compared to normal search on the same database. Moreover, it takes almost only 4% running time (21,219 sec. vs. 536,104 sec.) of the most widely used homology extension procedure (i.e., normal search on NCBI nr database), which is twenty-five times faster than the original required running time. Our results demonstrate that applying a fast search on a smaller compact database in homology extension can greatly reduce running time up to 96% without sacrificing prediction performance. This observation is useful for the community using homology extension, e.g., secondary structure prediction, MSAs, and etc. Therefore, the following analyses and experiments are performed based on UniRef50 with fast search model.

### 2. Interpretable visualization of feature reduction by correspondence analysis

**Figure 4:**
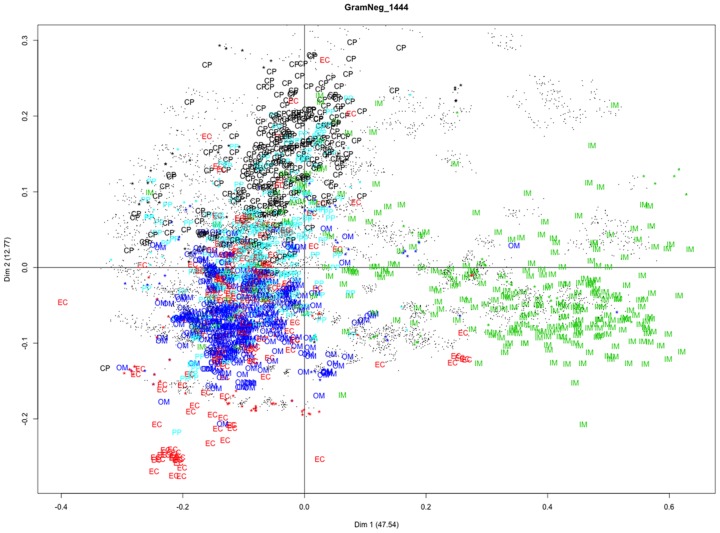
Correspondence analysis of the GramNeg_1444 data set. The figure shows Gram-negative bacteria proteins with localization labels, gapped-dipeptides (black circle) and gapped-dipeptide signatures (stars with corresponding color) projected in top two major CA dimensions whose percentage of variance is shown in parentheses.

Our experiment results demonstrate that feature reduction based on CA not only efficiently identify discriminative features, but also provides a more interpretable visualization for both protein localization and human kinase classification. For protein localization classification, we incorporate the GramNeg1444 data set as an example for comparison with PLSA feature reduction used in our previous PSLDoc study. Figure 4 illustrates the proteins represented by localization labels with different colors, and gapped-dipeptides denoted by dots and projected in top two major CA dimensions, whose percentages of variances are shown in brackets. The analysis of Figure 4 takes 1m29.294s in HP z800 workstation with Dual (2x) Intel Hex (6x) Core Xeon X5650 2.67 GHz and 47.3 GB memory. First, we examine the proteins labeled with localization classes with different colors in the plot. Interestingly, most of the localization sites are nicely separated in two major CA dimensions, which explain 60.31% of the variance in the data. However, the EC and OM classes tend to mix together, which can also reflect misclassifications between the EC and OM proteins in our method. Second, we analyze the correlation between the distribution of gapped-dipeptides and different localization classes in the plot. In CA, it is observed that some gapped-dipeptides stay closely with a specific localization class, which implies that these gapped-dipeptides show preference to the localization site. Gapped-dipeptide signatures proposed in PSLDoc are marked as stars in Figure 4, and their colors are assigned according to their corresponding preference localization sites (Table 7 in [6]). Interestingly, same colored gapped-dipeptide signatures and localization sites tend to cluster together. In other words, the preference relation between gapped-dipeptides and localization sites previously proposed in PSLDoc is consistent with CA. Now, this preference relation can be efficiently identified in one step by CA instead of the previous complex procedures in PLSA (Figure 8, 9 and 10 in [6]). Besides efficiency, CA also provides a more intuitive visualization of the preference relation.

**Figure 5:**
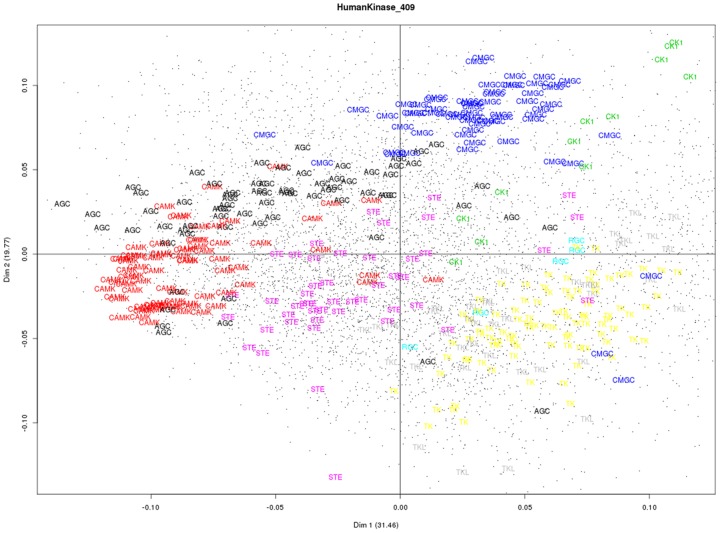
Correspondence analysis of the HumanKinase_409 data set. The figure shows human kinase sequences with family labels and gapped-dipeptides (black circle) projected in top two major CA dimensions whose percentage of variance is shown in parentheses.

For human kinase classification, Figure 5 shows human proteins represented by kinase family labels in and gapped-dipeptides projected in top two major CA dimensions for the HumanKinase_409 data set. We can observe a better separation in CA, which is consistent with a high overall prediction accuracy of 99.02%. We also use CA to further classify each human ePKs family into sub-families as defined in Manning *et al*. [36]. Figure S1 in Supporting Information gives an overview of the results we obtain for the sub-family classification of the AGC family.

### 3. Combination of precise prediction by compact sets and interpretable results from nearest neighbor algorithm

In our study, if a target protein belongs to compact sets, the final prediction is made by CS; otherwise, *1*−NN is used to assign the functional class. We utilize protein localization and kinase classification benchmark data sets for comparison with other approaches. For protein localization classification, Table 2 shows the prediction performance based on CS, CS+*1*−NN, and the original PSLDoc for the GramNeg_1444 data set. It is observed that CS only predicts 870 of 1,444 proteins but a precision of 100% is achieved. This demonstrates that the neighboring relation identified by CS is highly reliable. Furthermore, we incorporate a simple *1*−NN to predict the remaining “unknown” proteins based on CS (i.e., the CS+*1*−NN method) and achieve an overall recall of 91.97%, which is comparable to the prediction performance made by SVM-based predictors, such as the 91.60% by CELLO II [1] (recall and precision not available for comparison). To compare with the state-of-the-art system PSORTb 3.0, we also incorporate three new data sets built in PSORTb 3.0 for prediction performance evaluation.

Table 3 shows the prediction performance for the Gram-negative, Gram-positive, and Archaeal data sets. It is also observed that CS method reaches 100% precision with low recall for the three data sets. Poor recall in CS method is resulted from the limitation that CS method can only predict the proteins belonged to a compact set. On the other hand, *1*−NN method attains much higher recall because it can be applied to classify any protein in a data set. Most importantly, when CS and *1*−NN are combined together, experiment results demonstrate that CS+*1*−NN method attains a comparable performance with PSORTb 3.0 for both recall and precision. For the three data sets, we can observe that our CS+*1*−NN method reaches higher recall while PSORTb 3.0 obtains better prediction. Moreover, when both recall and precision are considered, our method achieves higher in F-measure at 0.9606 and 0.9689 compared to 0.9567 and 0.9526 by PSORTb 3.0 for the GramNeg_8230 and Archaeal_810 data sets, respectively. This indicates that our method can provide a more efficient and interpretable mean to predict protein localization without sacrificing discrimination power.

Table 4 shows the performance comparison of CS, CS+*1*−NN, and the state-of-the-art system, Kinomer, for the HumanKinase_409 data set. The precision of proteins predicted by CS is also 100% under a 71.39% recall (i.e., 292 proteins predicted). This corresponds well with our previous observation that identification of neighboring relations by CS is highly reliable. Moreover, when a simple *1*−NN is applied to classify the remaining unknown proteins from CS, the precision and recall are enhanced to 99.02% and 99.02%, respectively. Based on a compact set and simple *1*−NN, our prediction method can achieve comparable performance that is only slightly worse than the state-of-the-art recall at 99.77%, which could be overestimated because it was trained and tested on the same set instead of five-fold cross-validation in an HMM method by Kinomer.

## Discussion and Conclusions

First, for feature extraction and representation, evolutionary information in the PSSM has been commonly used for protein structure and function prediction, such as PSL prediction, function classification, and secondary structure prediction. Most methods generated PSSM information by searching against the NCBI nr database [38]. It has been shown that highly non-redundant UniRef databases can be used to obtain comparable prediction performance with NCBI nr in around one fourth of the running time in MSAs [20]. In this study, experiment results correspond well with the above observation that applying a fast search on a more compact non-redundant data set in homology extension can greatly increase efficiency without sacrificing prediction performance for both localization and kinase classification. Most notably, it would be helpful for researches in the bioinformatics community to consider this finding when they try to develop programs or web servers that can make accurate prediction in much less running time.

As for feature reduction by CA, our results demonstrate that proteins with different functional categories can be nicely separated by CA for both protein localization and human kinase classification. For localization data sets, experiment results show that CA can not only discriminate proteins from different localization sites but also provide a more efficient and visualized feature reduction compared with PLSA. The separation between proteins from varied kinase families in CA is especially clear for the human kinase data set, which lend support on the assumption that different functional classes can be accurately distinguished if more discriminative features can be proposed after feature reduction. Besides gapped-dipeptides, many sequence features have also been reported effective for protein classification, i.e., *k*-mer [39], class frequency [40], and etc. Due to the generality of our method, they can be incorporated into our framework such that the preference of these features with respect to the protein classes can be identified through CA in the future.

For the machine learning approaches, we propose a hybrid method in which CS is incorporated if a target protein belongs to compact sets; otherwise, the final functional class is predicted by *1*−NN. We propose CS to identify neighboring relation of proteins with high precision and experiment results indicate that proteins belonged to the same compact sets tend to have an identical proteins class. In addition, we apply *1*−NN because it is a simple and intuitive prediction method compared with SVM, and *k* is chosen as 1 (*k* = 1 in *k*-NN) to avoid overestimation in parameter tuning. Our study demonstrates that the combination of CS and *1*−NN is able to generate interpretable prediction results and achieve comparable performance compared with the state-of-the-art systems for both protein localization and human kinase classification.

The ability to correctly classify protein kinases into their respective families without the need of time consuming MSAs represents one of the major benefits of our method. While classifying the human ePKs may not be seen as challenging, one shall keep in mind that we designed our method for the task of classifying non-annotated proteins from species evolutionarily distant from common model species. Indeed, when analyzing the ePKs from orphan species, one may encounter sever difficulties to accurately align their sequences with the annotated ones. Thus, a method able to classify a set of distant sequences without the need of a MSA may be able to cover the gap left by the alignment based procedure. We believe that a meta-classifier based on both approaches should be able to deal with challenging classifications thanks to the use of two orthogonal signals. The source codes to generate gapped-dipeptides weighted by TFPSSM scheme and find compact sets are available at https://github.com/warnname/PSLDoc and https://github.com/warnname/FCS, respectively.

## Supporting Information

Figure S1 **Correspondence analysis for kinase sublabels of AGC family.** The figure shows human kinase sequences in AGC family with subfamily labels projected in top two major CA dimensions.
(DOC)

Text S1 **Proof of compact sets.**
(DOC)
