# Supplementary figures and images for: Efficient and Interpretable Prediction of Protein Functional Classes by Correspondence Analysis and Compact Set Relations
Source: PLoS One. 2013 Oct 11;8(10):e75542. doi: 10.1371/journal.pone.0075542 (PMC3795737; doi:10.1371/journal.pone.0075542)

### Figure S1 – Correspondence analysis for kinase sublabels of AGC family


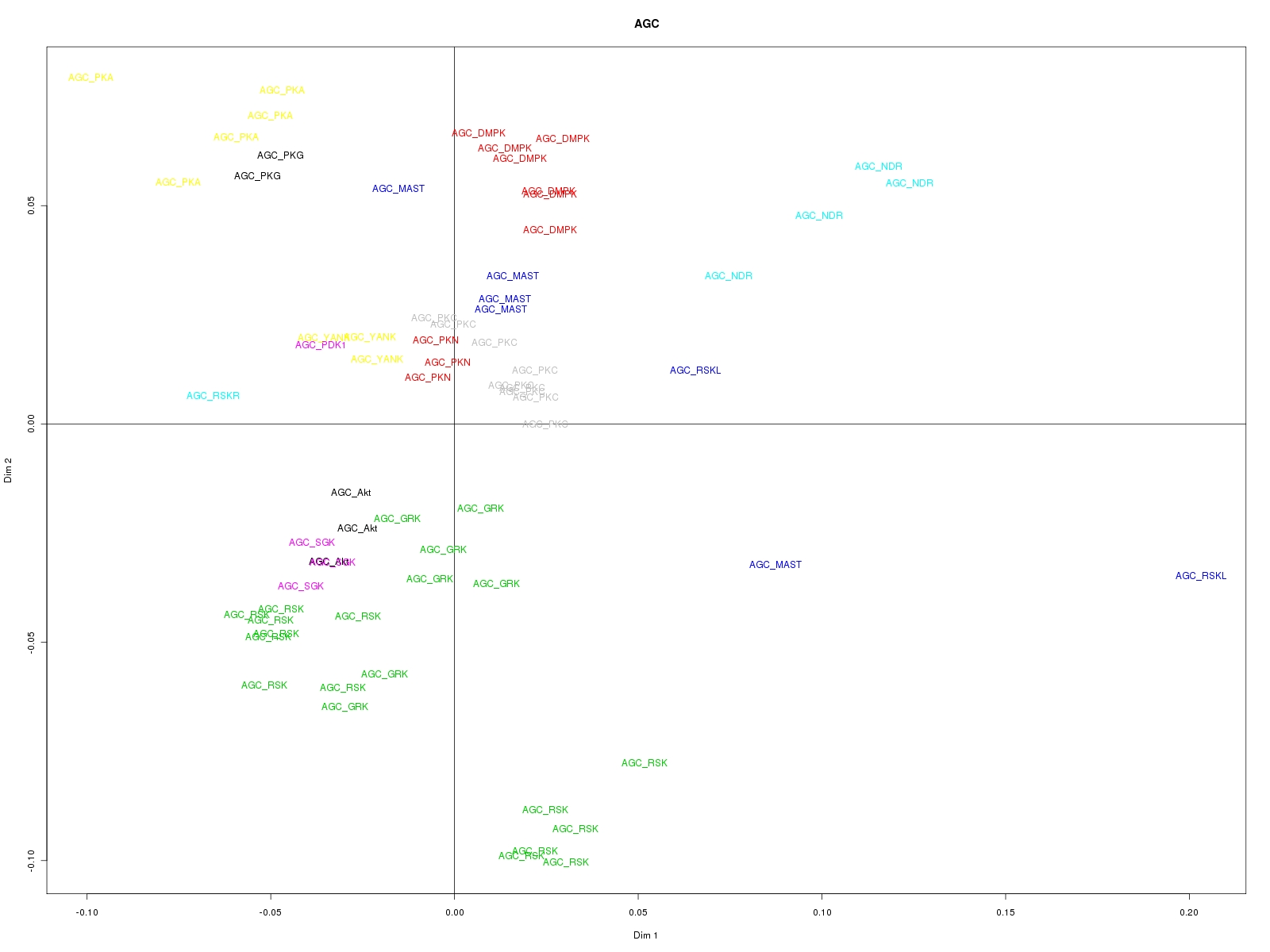

Supplement: Figure S1 — Correspondence analysis for kinase sublabels of AGC family. The figure shows human kinase sequences in AGC family with subfamily labels projected in top two major CA dimensions. (DOC) [file pone.0075542.s001.doc]
