# Supplementary material for: Efficient and Interpretable Prediction of Protein Functional Classes by Correspondence Analysis and Compact Set Relations
Source: PLoS One. 2013 Oct 11;8(10):e75542. doi: 10.1371/journal.pone.0075542 (PMC3795737; doi:10.1371/journal.pone.0075542)
Supplement: Text S1 — Proof of compact sets. (DOC) [file pone.0075542.s002.doc]

### Text S1 – Proof of compact sets

**Theorem 1.** *T_Kru_* is the same as *T_can_* [19, 20].

**Proof.** Without the loss of generality, we suppose that a graph *G* contains edges without equal lengths. Let *E_can_* be a set of sorted edges from a minimum spanning tree *T_sp_* in non-increasing order (as mentioned in Figure 1, [20]) and *E_kru_* be a set of edges that make if-condition in Algorithm CONSTRUCT_*T_Kru_* true. Clearly, both *E_can_* and *E_kru_* are the sets of edges of *T_sp_* of same graph *G*. Hence, *E_kru_* is equal to *E_can_*. *T_Kru_* is constructed by merging species according to edges of *E_kru_* in increasing order. If we deconstruct *T_Kru_* in the opposite direction, it is the same with splitting species according to edges of *E_can_* in non-increasing order. So, *T_Kru_* is the same as *T_can_*.
